# 

**Published:** 1865-10

**Authors:** 


### CONTENTS.

ORIGINAL ESSAYS AND COMMUNICATIONS.

Influence of the Viscera of the Human Body upon the Dental Organs,........ 395
Essay read before the Iowa State Dental Association,........ 398
Acquirements for admission into the Dental profession,........ 402
Sharp Sticks,........ 404

PROCEEDINGS OF SOCIETIES.

Proceedings of the Iowa State Dental Society........ 406

SELECTIONS.

Nitrous Oxide,........ 433
Phosphor-Necrosis,........ 436
Medical Schools,........ 437
Medical Department of the Army,........ 437
Resolutions of the Mad River Valley Dental Association,........ 438

EDITORIAL.

The Ohio College of Dental Surgery,........ 439
On Teething of Infants,........ 439
Researches on the Medical Properties and Applications of Nitrous Oxide, Protoxide of Nitrogen or Laughing Gas,........ 440

